# Supplementary material for: Decrease in the Size of Fat-Enlarged Axillary Lymph Nodes and Serum Lipids after Bariatric Surgery
Source: Cells. 2022 Jan 30;11(3):482. doi: 10.3390/cells11030482 (PMC8834314; doi:10.3390/cells11030482)
Supplement: Supplementary file 1 [file cells-11-00482-s001.zip › cells-1481278-supplementary.pdf]

The supplementary data provided in tables S1 and S2 include data for the entire bariatric surgery cohort that had available screening mammograms before and after surgery, including patients with partially visualized nodes, non-visualized nodes, and normal nodes that were not included in the primary analyses. Table S1 compares BMI and weight before and after bariatric surgery among all groups stratified by lymph node category (Supplementary Table S1). Table S2 provides detailed pre and post-surgical mean cardiometabolic profile of all groups stratified by lymph node category.

Supplementary Table S1. Study population characteristics by subgroup. *p*-values for continuous and categorical variables were calculated by a two-tailed *t*-test and a two-tailed Fisher's exact test, respectively. Missing values (NA) represent measurements of FIN that could not be obtained because the lymph nodes were not fully visible or were normal in size and morphology before and after bariatric surgery.

	Groups					
	**No Change in FIN Size**	**Change in FIN Size**	**Partially Visualized FIN**	**No Nodes Visualized**	**Normal Nodes**	***p*-Value**
n	39	45	40	21	10	
Age in years mean(sd)	56.26 (7.50)	58.40 (7.10)	54.62 (8.21)	55.00 (7.91)	57.20 (13.72)	0.259
Lymph node size in mm mean (sd)						
Pre-surgical	20.43 (6.17)	26.74 (8.81)	NA	NA	NA	<0.001
Post-surgical	18.65 (6.45)	20.37 (7.06)	NA	NA	NA	0.252
Difference	1.77 (1.15)	6.38 (5.06)	NA	NA	NA	<0.001
BMI kg/m^2 mean (sd)						
Pre-surgical	44.20 (7.61)	46.55 (8.14)	45.61 (5.75)	41.83 (5.71)	42.65 (6.15)	0.087
Post-surgical	34.60 (6.05)	34.07 (6.43)	32.82 (5.15)	32.10 (7.26)	33.14 (6.59)	0.526
Difference	9.60 (5.30)	12.48 (6.14)	13.61 (7.15)	9.73 (3.70)	9.51 (1.75)	0.009
Weight in kg mean (sd)						
Pre-surgical	116.97 (21.09)	122.56 (24.02)	123.60 (17.85)	112.64 (16.54)	113.88 (16.89)	0.182
Post-surgical	91.26 (16.93)	89.54 (17.24)	86.44 (16.33)	85.46 (20.22)	85.76 (14.61)	0.62
Difference	25.71 (14.28)	33.02 (16.94)	37.16 (23.44)	27.19 (8.77)	28.12 (6.47)	0.031

Supplementary Table S2. Detailed cardiometabolic profile of all patients before and after bariatric surgery stratified by fat-infiltrated lymph node category. Body mass index (BMI) in kg/m^2, BP (blood pressure) in mmHg, Hba1c (hemoglobin A1c) in mg/dL, HDL (high-density lipoprotein) in mg/dL, LDL (low-density lipoprotein) in mg/dL, TG (triglyceride) in mg/dL, and Chol (cholesterol) in mg/dL. *p*-values were calculated by a two-tailed *t*-test.

	**Pre-Surgery**	**Post-Surgery**	***p* Value**
Change in FIN size			
n	45	45	
BMI (mean (SD))	46.55 (8.14)	34.07 (6.43)	<0.001
Weight (mean (SD))	122.56 (24.02)	89.54 (17.24)	<0.001
Systolic (mean (SD))	141.09 (14.95)	128.89 (14.91)	<0.001
Diastolic (mean (SD))	76.62 (10.82)	72.49 (11.37)	0.081
Fasting glucose (mean (SD))	132.21 (38.88)	102.38 (19.74)	0.002
Hba1c (mean (SD))	6.62 (1.15)	6.27 (1.17)	0.228

Chol (mean (SD))	198.54 (37.58)	187.76 (34.44)	0.291
HDL (mean (SD))	51.62 (16.00)	65.96 (18.32)	0.004
TG (mean (SD))	174.08 (100.07)	128.72 (68.86)	0.066
LDL (mean (SD))	112.12 (36.14)	96.58 (31.37)	0.112
No change in FIN			
n	39	39	
BMI (mean (SD))	44.20 (7.61)	34.60 (6.05)	<0.001
Weight (mean (SD))	116.97 (21.09)	91.26 (16.93)	<0.001
Systolic (mean (SD))	138.95 (11.19)	127.23 (14.20)	<0.001
Diastolic (mean (SD))	77.13 (12.80)	70.54 (10.42)	0.015
Fasting glucose (mean (SD))	125.75 (34.58)	107.06 (31.41)	0.103
Hba1c (mean (SD))	6.35 (0.89)	5.79 (1.10)	0.036
Chol (mean (SD))	215.80 (41.54)	192.85 (45.22)	0.103
HDL (mean (SD))	49.10 (10.97)	58.55 (15.68)	0.033
TG (mean (SD))	209.30 (107.13)	132.85 (51.86)	0.007
LDL (mean (SD))	124.45 (38.28)	108.55 (41.60)	0.216
Partially visible FIN			
n	40	40	
BMI (mean (SD))	45.61 (5.75)	32.82 (5.15)	<0.001
Weight (mean (SD))	123.6 (17.85)	86.44 (16.3)	<0.001
Systolic (mean (SD))	133.97 (11.98)	126.20 (14.21)	0.01
Diastolic (mean (SD))	75.92 (7.67)	68.58 (8.67)	<0.001
Fasting glucose (mean (SD))	112.47 (24.77)	98.57 (21.17)	0.101
Hba1c (mean (SD))	6.23 (1.08)	5.88 (0.86)	0.258
Chol (mean (SD))	195.11 (33.76)	177.35 (36.82)	0.146
HDL (mean (SD))	51.94 (14.24)	56.71 (13.88)	0.324
TG (mean (SD))	154.89 (96.80)	125.18 (39.45)	0.248
LDL (mean (SD))	113.28 (31.79)	95.59 (31.51)	0.108
No nodes visible			
n	21	21	
BMI (mean (SD))	41.83 (5.71)	32.10 (7.26)	<0.001
Weight (mean (SD))	112.64 (16.54)	85.45 (20.22)	<0.001
Systolic (mean (SD))	134.48 (13.66)	124.00 (15.06)	0.023
Diastolic (mean (SD))	76.00 (10.16)	74.14 (9.25)	0.539
Fasting glucose (mean (SD))	109.91 (20.44)	105.67 (36.07)	0.744
Hba1c (mean (SD))	5.85 (0.66)	5.43 (0.31)	0.093
Chol (mean (SD))	189.71 (38.59)	188.83 (19.09)	0.961
HDL (mean (SD))	47.14 (10.64)	56.33 (12.24)	0.175
TG (mean (SD))	145.57 (72.55)	112.17 (75.90)	0.435
LDL (mean (SD))	115.00 (27.74)	110.00 (18.57)	0.715
Normal nodes visible			
n	10	10	
BMI (mean (SD))	42.65 (6.15)	33.14 (6.59)	0.004
Weight (mean (SD))	113.88 (16.89)	85.76 (14.61)	0.001
Systolic BP (mean (SD))	133.60 (13.32)	131.90 (14.83)	0.79
Diastolic BP (mean (SD))	71.80 (12.61)	73.80 (8.51)	0.683
Fasting glucose (mean (SD))	88.17 (5.34)	92.20 (19.40)	0.634

Hba1c (mean (SD))	5.76 (0.87)	5.23 (0.42)	0.372
Chol (mean (SD))	215.00 (44.98)	198.67 (33.66)	0.481
HDL (mean (SD))	56.86 (10.65)	80.17 (9.54)	0.002
TG (mean (SD))	151.57 (79.83)	68.17 (8.13)	0.028
LDL (mean (SD))	120.33 (31.06)	105.00 (30.12)	0.406
